# Identifying resistance in wild and ornamental cherry towards bacterial canker caused by *Pseudomonas syringae*

**DOI:** 10.1111/ppa.13513

**Published:** 2021-12-21

**Authors:** Michelle T. Hulin, Andrea Vadillo Dieguez, Francesca Cossu, Samantha Lynn, Karen Russell, Helen C. Neale, Robert W. Jackson, Dawn L. Arnold, John W. Mansfield, Richard J. Harrison

**Affiliations:** ^1^ NIAB EMR East Malling UK; ^2^ NIAB Cambridge UK; ^3^ K Russell Consulting Ltd Huntingdon UK; ^4^ Centre for Research in Bioscience Faculty of Health and Applied Sciences The University of the West of England Frenchay Campus Bristol UK; ^5^ Birmingham Institute of Forest Research (BIFoR) University of Birmingham Birmingham UK; ^6^ School of Biosciences University of Birmingham Birmingham UK; ^7^ Harper Adams University Newport Shropshire UK; ^8^ Faculty of Natural Sciences Imperial College London London UK; ^9^ Present address: The Sainsbury Laboratory Norwich UK

**Keywords:** disease resistance, *Pseudomonas syringae*, tree disease

## Abstract

Bacterial canker is a major disease of stone fruits and is a critical limiting factor to sweet cherry (*Prunus avium*) production worldwide. One important strategy for disease control is the development of resistant varieties. Partial varietal resistance in sweet cherry is discernible using shoot or whole tree inoculations; however, these quantitative differences in resistance are not evident in detached leaf assays. To identify novel sources of resistance to canker, we used a rapid leaf pathogenicity test to screen a range of wild cherry, ornamental *Prunus* species and sweet cherry × ornamental cherry hybrids with the canker pathogens, *Pseudomonas syringae* pvs *syringae*, *morsprunorum* races 1 and 2, and *avii*. Several *Prunus* accessions exhibited limited symptom development following inoculation with each of the pathogens, and this resistance extended to 16 *P*. *syringae* strains pathogenic on sweet cherry and plum. Resistance was associated with reduced bacterial multiplication after inoculation, a phenotype similar to that of commercial sweet cherry towards nonhost strains of *P*. *syringae*. Progeny resulting from a cross of a resistant ornamental species *Prunus* *incisa* with susceptible sweet cherry (*P*. *avium*) exhibited resistance indicating it is an inherited trait. Identification of accessions with resistance to the major bacterial canker pathogens is the first step towards characterizing the underlying genetic mechanisms of resistance and introducing these traits into commercial germplasm.

## Graphical abstract

This research paper identifies resistance towards *Pseudomonas* bacterial canker in *Prunus* species using detached leaf assays.

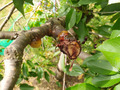

## 1 INTRODUCTION

Plant diseases caused by bacteria remain problematic for the global horticultural industry due to a lack of effective control measures (Sundin et al., 2016). The genus *Prunus* contains over 400 species, a selection of which are grown for top fruit, ornamental use and timber production (Bortiri et al., 2001). Bacterial canker, caused by members of the *Pseudomonas syringae* species complex, can be a major limiting factor in the cultivation of *Prunus* spp. (Omrani et al., 2019; Vicente et al., 2004). The disease is primarily characterized by necrosis, gummosis and/or dieback of woody plant tissues. In addition, the pathogens colonize other plant tissues where they exist epiphytically or invade to cause leaf and fruit spots, and blossom blight. These tissues can act as reservoirs for later woody tissue infection (Crosse, 1966). At least five phylogenetically distinct clades of *P*. *syringae* are known to cause bacterial canker on *Prunus*. These include *P*. *syringae* pv. *morsprunorum* race 1 (Psm R1), *P*. *syringae* pv. *morsprunorum* race 2 (Psm R2), *P*. *syringae* pv. *syringae* (Pss), *P*. *syringae* pv. *persicae* and the more recently discovered *P*. *syringae* pv. *avii* (Psa), and *P*. *cerasi* (Kałużna et al., 2016; Ménard et al., 2003; Parisi et al., 2019). Psm R1 and Psm R2 are genetically distinct, belonging to different phylogroups within the species complex and can be alternatively referred to as within the species *P*. *amygdali* and *P*. *avellanae*, respectively (Gomila et al., 2017). Bacterial strains differ in host range and aggressiveness towards particular species in the genus (reviewed in Bultreys & Kałużna, 2010). A recent study identified a range of factors that contributed to bacterial virulence, but also found that knockout of genes encoding possible avirulence proteins, including the effector *HopAU1*, led to hypervirulent bacterial phenotypes, suggesting a quantitative level of resistance exists even in susceptible cultivars (Neale et al., 2021).

Control measures available for bacterial canker are limited. The genotypically diverse *P*. *syringae* clades causing the disease may vary in sensitivity to control measures and can rapidly evolve and transfer genes conferring resistance to chemicals such as copper‐based biocides and antibiotics (Sundin et al., 2016). The genetic diversity of bacterial canker pathogens poses a challenge in the generation of novel controls because responses to all potential pathogens must be tested. Progress is being made in the development of specific biological controls such as the use of bacteriophages (Rabiey et al., 2020) that could be used in combinations effective against all clades. A complementary approach is to breed for resistance, a strategy particularly important in forestry, where spraying control is impractical (Vicente et al., 2004). Ideally, resistance against multiple clades would be most beneficial or an alternative strategy would be to stack resistance‐associated loci effective against the different clades into new varieties.

The molecular mechanisms involved in plant resistance towards bacterial pathogens, such as *P*. *syringae*, have been extensively characterized in model plant species such as *Arabidopsis thaliana*. Resistance involves heightened immunity that occurs at the plant cell surface through receptor detection of pathogen‐associated molecular patterns, as well as the intracellular detection of pathogen virulence proteins (effectors) injected into plant cells. These two components of resistance are now known to be intrinsically linked (Ngou et al., 2021).

There is limited knowledge of resistance in *Prunus* towards bacterial canker pathogens. Cherry and apricot varieties with partial resistance to one or more of the pathogens have been identified using methods such as laboratory‐based shoot inoculations and field tree inoculations (Farhadfar et al., 2016; Hulin, Mansfield, et al., 2018; Omrani et al., 2019; Santi et al., 2004). In our previous study, we found that the partial resistance seen in woody tissue of certain cherry cultivars was not detected using detached leaf syringe‐infiltration assays (Hulin, Mansfield, et al., 2018). This partial resistance seen in woody tissues is probably quantitative, involving multiple alleles having small effects, with the most resistant varieties still succumbing to disease under favourable conditions. Although only partial, such resistance could be highly useful for *Prunus* breeding as it could reduce overall pathogen load in orchards as part of an integrated disease management approach (Sundin et al., 2016). In addition, it is arguably more durable than single resistance (*R*) gene‐based immunity, which is, theoretically, more frequently overcome during pathogen evolution (Pilet‐Nayel et al., 2017). Progress towards understanding the genetic factors involved in bacterial canker resistance has been made by Omrani et al. (2019) who identified quantitative trait loci (QTLs) involved in partial resistance in apricot. These loci contain genes involved in phytohormone signalling, a process known to play a pivotal role during the plant immune response.

Studies reporting the screening of *Prunus* for canker resistance have focused on established commercial varieties. However, wild relatives can provide robust sources of disease resistance not found in crop genotypes and may be introduced during crop breeding. Nonhost resistance is defined as the ability of all genotypes of a plant species to resist all genotypes of a pathogen (Heath, 2000). Such resistance traits can be transferred into crops. For example, relatives of apple such as *Malus* × *robusta* 5 and *Malus floribunda* have been used extensively to introduce complete resistance towards the fireblight pathogen *Erwinia amylovora*, both through breeding and transgenic strategies (Campa et al., 2019). In addition, wild accessions of kiwifruit have been identified with resistance towards the canker pathogen *P*. *syringae* pv. *actinidiae* using large‐scale in vitro assays (Wang et al., 2020). *Prunus* is a diverse genus that includes five subgenera: *Amygdalus*, *Cerasus*, *Prunus*, *Laurocerasus* and *Padus* (Chin et al., 2014), with many natural and artificial interspecific hybrids. The subgenus *Cerasus* includes *P*. *avium* (sweet and wild cherry), *P*. *cerasus* (sour cherry) and *P*. *mahaleb*. Wild cherry is native to Europe, Africa and western Asia (Miljković et al., 2019) and exhibits greater genetic diversity than sweet cherry (Avramidou et al., 2010), potentially including diversity in genes conferring resistance to pathogens.

Studies have already shown wild *Prunus* species to be important sources of resistance to pathogens such as plum pox virus (Decroocq et al., 2005). Therefore, in this study, we aimed to identify resistance in accessions of wild cherry. Sweet cherry cultivars are known to vary in their resistance towards bacterial canker disease under field conditions (Farhadfar et al., 2016; Mgbechi‐Ezeri et al., 2017), but no complete resistance has been reported. We screened a wide variety of wild cherry accessions and *Prunus* species related to cherry for resistance to the bacterial canker pathogens. We also screened several hybrids of susceptible sweet cherry crossed with ornamental species. Our results have identified potential sources of resistance to members of each of the pathogenic clades of *P*. *syringae*.

## 2 MATERIALS AND METHODS

### 2.1 Plant material

The *Prunus* germplasm used in this study (Table 1) was propagated at the National Institute of Agricultural Botany East Malling Research (NIAB EMR), in East Malling, UK. The experiments conducted with each accession are listed in Table 1. Samples from mature trees, grown in fields at East Malling, were used for large screens including the sweet cherry shoot tests and leaf symptom screens of all wild, ornamental and hybrid *Prunus*. For tests of in planta bacterial multiplication in which material was needed for multiple repetition of experiments, selected accessions (*P*. *incisa*, Groton A, Groton B, Penny and Sweetheart), were grafted onto Gisela 5 rootstocks and actively growing 4‐month‐old trees were grown in polytunnels, to obtain leaves over an extended period. Due to limited leaf availability, either cultivars Penny or Sweetheart were used as sweet cherry susceptible controls in population counts.

**TABLE 1:** *Prunus* accessions screened in this study, including subgenus, species and accession

Subgenus	Species	Accession	Group	Abbreviation^a^	Experiment^b^
*Cerasus*	*P. avium*	Penny	Sweet	53	acefgi
*Cerasus*	*P. avium*	Sweetheart	Sweet	54	abcefh
*Cerasus*	*P. avium*	Van	Sweet	55	abc
*Cerasus*	*P. avium*	Colney	Sweet	56	abci
*Cerasus*	*P. avium*	Kordia	Sweet		a
*Cerasus*	*P. avium*	Merchant	Sweet		a
*Cerasus*	*P. avium*	Stella	Sweet		a
*Cerasus*	*P. avium*	Merton Glory	Sweet		ai
*Cerasus*	*P. avium*	Regina	Sweet		a
*Cerasus*	*P. avium*	Lapins	Sweet		a
*Cerasus*	*P. avium*	Roundel	Sweet		a
*Cerasus*	*P. avium*	Newstar	Sweet		a
*Cerasus*	*P. avium*	Summersun	Sweet		a
*Cerasus*	*P. avium*	Korvic	Sweet		a
*Cerasus*	*P. avium*	Inge	Sweet		a
*Cerasus*	*P. avium*	Napoleon	Sweet	P. av Nap	ad
*Cerasus*	*P. avium*	P. a. Arger Fen A	Wild	1	c
*Cerasus*	*P. avium*	P. a. Arger Fen E	Wild	2	c
*Cerasus*	*P. avium*	P. a. Barming Lane	Wild	3	c
*Cerasus*	*P. avium*	P. a. Beardown Wood	Wild	4	c
*Cerasus*	*P. avium*	P. a. Buckland Wood 8	Wild	5	c
*Cerasus*	*P. avium*	P. a. Bunny Old Wood A	Wild	6	c
*Cerasus*	*P. avium*	P. a. Bunny Old Wood B	Wild	7	c
*Cerasus*	*P. avium*	P. a. Burghley Wood	Wild	8	c
*Cerasus*	*P. avium*	P. a. Chalky Road	Wild	9	c
*Cerasus*	*P. avium*	P. a. Charger	Wild	10	c
*Cerasus*	*P. avium*	P. a. Cherryhill Copse A	Wild	11	c
*Cerasus*	*P. avium*	P. a. Chisbury Wood 1905	Wild	12	c
*Cerasus*	*P. avium*	P. a. Cobtree	Wild	13	c
*Cerasus*	*P. avium*	P. a. Coed‐Felin‐Gat	Wild	14	c
*Cerasus*	*P. avium*	P. a. Coed‐y‐Stig	Wild	15	c
*Cerasus*	*P. avium*	P. a. Deadmans Wood	Wild	16	c
*Cerasus*	*P. avium*	P. a. Dean Wood 1918	Wild	17	c
*Cerasus*	*P. avium*	P. a. Everdon Stubbs B	Wild	18	c
*Cerasus*	*P. avium*	P. a. FD1‐57‐4/122	Wild	19^d^	c
*Cerasus*	*P. avium*	P. a. Ffynone	Wild	20	c
*Cerasus*	*P. avium*	P. a. Frydd Wood 1908	Wild	21	c
*Cerasus*	*P. avium*	P. a. Groton A	Wild	22^c^	cef
*Cerasus*	*P. avium*	P. a. Groton B	Wild	23^c^, ^d^	cefgi
*Cerasus*	*P. avium*	P. a. Hamlet Wood C	Wild	24	c
*Cerasus*	*P. avium*	P. a. Howley Wood	Wild	25	c
*Cerasus*	*P. avium*	P. a. Lockeridge B	Wild	26	c
*Cerasus*	*P. avium*	P. a. Lowdham Lane	Wild	27	c
*Cerasus*	*P. avium*	P. a. Lower Broxford Wood A	Wild	28	c
*Cerasus*	*P. avium*	P. a. Lower Broxford Wood B	Wild	29	c
*Cerasus*	*P. avium*	P. a. Malvern Hills	Wild	30	c
*Cerasus*	*P. avium*	P. a. Marlow Common 1902	Wild	31	c
*Cerasus*	*P. avium*	P. a. Narth A	Wild	32	c
*Cerasus*	*P. avium*	P. a. Orleans−141	Wild	33	c
*Cerasus*	*P. avium*	P. a. Pencelli Wood B	Wild	34	c
*Cerasus*	*P. avium*	P. a. Penley Wood A	Wild	35	c
*Cerasus*	*P. avium*	P. a. Postlebury B	Wild	36	c
*Cerasus*	*P. avium*	P. a. Poulton Wood A	Wild	37	c
*Cerasus*	*P. avium*	P. a. Primrose Wood	Wild	38	c
*Cerasus*	*P. avium*	P. a. Prospect Cottage	Wild	39	c
*Cerasus*	*P. avium*	P. a. Roundhill Wood	Wild	40	c
*Cerasus*	*P. avium*	P. a. Saxtens Wood B	Wild	41	c
*Cerasus*	*P. avium*	P. a. SC 311–33 (S27, S28)	Wild	42	c
*Cerasus*	*P. avium*	P. a. Snarkhurst	Wild	43	c
*Cerasus*	*P. avium*	P. a. South Wood	Wild	44	c
*Cerasus*	*P. avium*	P. a. Stoke Row 1903	Wild	45	c
*Cerasus*	*P. avium*	P. a. Tank Wood	Wild	46	c
*Cerasus*	*P. avium*	P. a. Thornes Wood	Wild	47	c
*Cerasus*	*P. avium*	P. a. Thruxton Vallets	Wild	48^d^	c
*Cerasus*	*P. avium*	P. a. Thundersley Wood	Wild	49	c
*Cerasus*	*P. avium*	P. a. Tyn‐y‐Bryn	Wild	50	c
*Cerasus*	*P. avium*	P. a. Wepre Park	Wild	51	c
*Cerasus*	*P. avium*	P. a. Wilmay Copse	Wild	52	c
*Cerasus*	*P. avium*	Tetraploid	Ornamental	P. av 4×	d
*Cerasus*	*P. canescens*	F1296	Ornamental	P. cn F1	d
*Cerasus*	*P. canescens*	F1327	Ornamental	P. cn F2	d
*Cerasus*	*P. cerasus*	Kelleris 16	Ornamental	P. ce K16	d
*Cerasus*	*P. cerasus*	Ujfehertoi Furtos	Ornamental	P. ce UF	d
*Cerasus*	*P. dawyckensis*	GM61	Ornamental	P. da GM61	d
*Cerasus*	*P. incisa*	E621	Ornamental	P. in E621^c^, ^d^	defgh
*Cerasus*	*P. maackii*	G280	Ornamental	P. mc G280	d
*Cerasus*	*P. mahaleb*	SL64	Ornamental	P. mh SL64	d
*Cerasus*	*P. maximoriczii*		Ornamental	P. mx	d
*Cerasus*	*P. pennsylvanica*		Ornamental	P. pen^d^	d
*Cerasus*	*Prunus* sp.	Ingram Dwarf	Ornamental	P. sp. ID	d
*Cerasus*	*P*. × *gondouinii*	Kanzas Sweet	Ornamental	P × g KS	d
*Cerasus*	*P*. × *gondouinii*	Marvel Duke	Ornamental	P × g MD^d^	d
*Cerasus*	*P. cerasus*	Elmer	Ornamental	P. ce Elmer	d
*Cerasus*	*P. avium* × *P. canescens*	Napoleon × *P*. *canescens* F1327	Hybrid	Nap × P. cn F2^e^	d
*Cerasus*	*P. avium* × *P. kurilensis*	Napoleon × *P*. *kurilensis* (1)	Hybrid	Nap × P. ku(1)^e^	d
*Cerasus*	*P. avium* × *P. kurilensis*	Napoleon × *P*. *kurilensis* (2)	Hybrid	Nap × P. ku(2)^e^	d
*Cerasus*	*P. avium* × *P. nipponica*	Napoleon × *P*. *nipponica*	Hybrid	Nap × P. ni^e^	d
*Cerasus*	*P. avium* × *P. incisa*	Napoleon × *P*. *incisa* E621 (1)	Hybrid	Nap v P. in(1)^d,e^	d
*Cerasus*	*P. avium* × *P. incisa*	Napoleon × *P*. *incisa* E621 (2)	Hybrid	Nap × P. in(2)^d,e^	d
*Cerasus*	*P. avium* × *P. incisa*	Napoleon × *P*. *incisa* E621 (3)	Hybrid	Nap × P. in(3)^d,e^	d
*Cerasus*	*P. canescens* × *P. avium*	*P*. *canescens* F1296 × Napoleon	Hybrid	P. cn F1 × Nap^e^	d
*Cerasus*	*P. mahaleb* × *P. avium*		Hybrid	P. mh × P. av^e^	d
*Prunus*	*P. armeniaca*	Tomcot	*Prunus* sp.	P. ar	d
*Prunus*	*P. cerasifera*	M3	*Prunus* sp.	P. cf M3^d^	d
*Prunus*	*P. cerasifera*	M5	*Prunus* sp.	P. cf M5	d
*Prunus*	*P. cerasifera*	M7	*Prunus* sp.	P. cf M7	d
*Prunus*	*P. domestica*	Seneca	*Prunus* sp.	P. do Se	d
*Prunus*	*P. domestica*	Victoria	*Prunus* sp.	P. do Vic	d
*Amygdalus*	*P. amygdalo‐persica*	MB137 2817	*Prunus* sp.	P. a‐p	d
*Amygdalus*	*P. dulcis*	Redwood	*Prunus* sp.	P. du RW^d^	d
*Amygdalus*	*P. persica*	Hiu Hun Tao	*Prunus* sp.	P. per	d
*Padus*	*P*. *padus* × Virginia	C292‐2	*Prunus* sp.	P. pad × Vir^d^	d

^a^ Abbreviation used on Figures 3 and 4.

^b^ Experiments in which each accession is included: a: sweet cherry cut‐shoot assay of susceptibility (Figure 1, Figure S1), b: bacterial populations in sweet cherry leaf after inoculation with different inoculum concentrations (Figure 2), c: screen of symptoms in wild cherry leaves (Figure 3), d: screen of symptoms in other *Prunus* species leaves (Figure 4), e: large leaf symptom screen of selected accessions with 16 bacterial strains (Figure 5), f: bacterial population counts in leaves of selected accessions (Figure 6), g: selected accession leaf bacterial population counts at different inoculum concentrations (Figure 7), h: leaf bacterial population count with nonhost *P*. *syringae* strains (Figure 8), i: cut‐shoot inoculation with selected accessions (Figure 9).

^c^ Accessions tested further after initial screening.

^d^ Accessions showing significantly reduced symptom development compared to susceptible sweet cherry controls.

^e^ *Prunus* subgenus *Cerasus* interspecific hybrids from crosses with *P*. *avium*.

Sixteen sweet cherry cultivars were examined in cut‐shoot inoculation tests and a subset was also used for detached leaf assays. Fifty‐two genotypes of wild cherry (*P*. *avium*) were screened with detached leaf assays. These included trees originally propagated from woodland across the United Kingdom (GPS coordinates are listed in Table S1), intentionally representing the nationwide diversity of this species and focusing on accessions of interest for the forestry industry. In addition, 34 relatives of sweet cherry were included in the detached leaf screening programme. These relatives included 15 ornamental species/known hybrids within the subgenus *Cerasus*, nine interspecific hybrids (susceptible sweet cherry cv. Napoleon crossed with the ornamental species *P*. *canescens*, *P*. *incisa*, *P*. *nipponica*, *P*. *kurilensis* and *P*. *mahaleb*), as well as 10 accessions of additional *Prunus* species from different subgenera (*Amygdalus*, *Prunus* and *Padus*).

### 2.2 Bacterial strains

Strains of *P*. *syringae* used and the experiments they were included in are listed in Table S2. The most used strains were Psm R1‐C (R1‐5244), originally isolated from sweet cherry; Psm R1‐P (R1‐5300), isolated from plum with low virulence on sweet cherry; Psm R2 (PsmR2‐leaf, renamed MH001), isolated from sweet cherry; and Pss (Pss 9644), also isolated from sweet cherry. For the wild cherry screening, the pathogen *P*. *syringae* pv. *avii* (avii5271) was also included. Screening was later extended to a diverse range of strains on selected *Prunus* accessions. The pathogenicity of the strains was extensively characterized in Hulin, Mansfield, et al. (2018). Culturing and inoculum preparation were as described in this previous work (Hulin, Mansfield, et al., 2018). Briefly, strains obtained from long‐term 20% glycerol stocks held at −80°C were grown on King's B agar (King et al., 1954) for 2–3 days at 28°C. Single colonies were then inoculated into lysogeny broth and grown overnight at 28°C with orbital shaking at 180 rpm. Cultures were centrifuged at 3500 × *g* for 10 min before resuspending in 10 mM MgCl_2_ to an optical density at 600 nm (OD_600_) of 0.2, which corresponds to approximately 2 × 10^8^ cfu/ml. This inoculum was then diluted to generate the different inoculum concentrations required for each experiment.

### 2.3 Pathogenicity assays

Shoots were collected from mature trees and inoculated using the dip inoculation method described in Hulin, Mansfield, et al. (2018). Briefly, 1‐year‐old shoots, 12 cm in length, were collected from field‐grown trees when dormant (December–February). Before inoculation, shoots were surface sterilized with 70% ethanol and allowed to air dry. The apical end was cut with secateurs (removing 1 cm) and dipped in bacterial inoculum of 2 × 10^7^ cfu/ml for 5 min. Shoots were blotted dry on paper towel and sealed with Parafilm. The basal end of the shoot was then cut (removing 1 cm) and the shoot was kept in water for 1 week at 16°C with 16:8‐h light:dark cycles. Shoots were then placed in Oasis foam in a fully randomized design and kept at 16°C in a controlled environment room for a further 5 weeks with 16:8 h light:dark cycles. They were routinely watered to keep the foam constantly moist. At the end of the experiment, shoots were assessed by peeling away the top layer of tissue and measuring the length of underlying necrosis. This experiment was repeated five times.

Detached leaf pathogenicity assays were conducted in spring 2018 and 2020, using 2‐ to 3‐week‐old leaves from field‐grown mature trees. For population counts, leaves from actively growing 4‐month‐old grafted trees in polytunnels were used to allow multiple repetitions of these experiments. The top three fully expanded leaves were chosen for experiments, due to their expected similar susceptibility (Mgbechi‐Ezeri et al., 2017).

Leaf pathogenicity assays and population counts were conducted as in Hulin, Mansfield, et al. (2018). Leaves were infiltrated using a blunt‐ended syringe with inoculum usually at a concentration of 2 × 10^6^ cfu/ml (100‐fold dilution of a 0.2 OD_600_ suspension). After incubation for 10 days at 22°C, this concentration of inoculum allowed clear differentiation between responses to strains pathogenic to cherry and to other hosts (Hulin, Mansfield, et al., 2018). Each leaf received a mock inoculation as a control and, where appropriate, different strains were compared on the same leaves (up to six inoculation sites) to reduce plant variability. Symptoms were scored on a scale of 0–5 (0, none; 1, limited browning; 2, browning <50% inoculated area; 3, browning >50% inoculated area; 4, complete browning; 5, complete browning with spread away from initial lesion). Experiments were repeated at least three times. Population counts of bacteria within disease lesions were conducted as previously described (Hulin, Mansfield, et al., 2018): leaves were surface sterilized with 70% ethanol before excision of leaf disks from the inoculated area with a 0.5 cm cork‐borer and ground in 10 mM MgCl_2_. Serial dilutions were plated onto King's B agar with cephalexin (80 mg/L) and cycloheximide (200 mg/L).

### 2.4 Statistical analysis

All statistical analyses and graph generation were performed using R software (R Core Team, 2012), and the packages ggplot2, lmerTest, lme4, emmeans, ordinal and multcomp (Bates et al., 2015; Christensen, 2019; Hothorn et al., 2008; Lenth et al., 2020; Wickham, 2009). For population counts and necrosis data from shoot experiments, analysis of variance (ANOVA) was used to determine statistical differences between treatments. Where data sets were unbalanced due to the grouping of multiple experiments with one or more treatments missing, REML was used to generate a linear mixed model. Means were extracted from the model using the program emmeans and post hoc comparisons generated using the cld function within the multcomp package. Where residuals from the linear model/ANOVA were not normally distributed, the data were log transformed and the model run again and residuals checked with qqnorm. To analyse the symptom score data from pathogenicity assays, the ordinal package was used, specifically the function clmm, which is optimized for ordinal data.

## 3 RESULTS

### 3.1 Partial resistance is seen in woody tissue but not leaf tissue of sweet cherry cultivars

**FIGURE 1:**
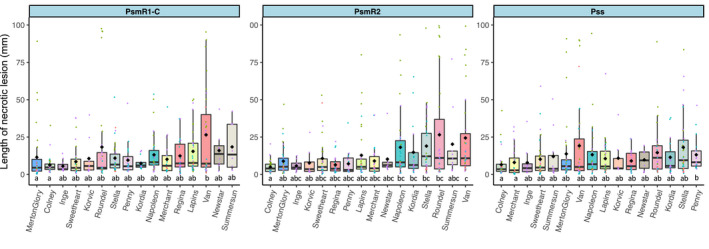
Susceptibility of sweet cherry cultivars to *Pseudomonas syringae* infection. Boxplots show length of necrotic lesions in cut‐shoots inoculated with *P*. *syringae* pv. *morsprunorum* race 1‐C (Psm R1‐C 5244), *P*. *syringae* pv. *morsprunorum* race *2* (Psm R2 MH001), or *P*. *syringae* pv. *syringae* (Pss 9644), 6 weeks after inoculation. The boxplots are ordered by estimated marginal means derived from the linear model to visualize the range of responses, although the graphs are of raw data. Individual data points are included and coloured for each separate experiment and the arithmetic mean is shown with a black diamond. This experiment was repeated up to five times per cultivar × strain combination. This figure shows the results for the three main pathogens, while the full data including results using Psm R1‐Plum and mock‐inoculated controls (neither of which caused significant symptoms) are presented in Figure S1. REML analysis indicated a significant difference between cultivars (*p* < 0.01, *df* = 15), strains (*p* < 0.01, *df* = 4) and an interaction between them *(p* < 0.01, *df* = 60). Tukey HSD (*p* = 0.05, confidence level: 0.95) significance groups obtained from the estimated marginal means (emmeans) are presented separately for each bacterial strain as letters under the graph

Varietal resistance has been reported in sweet cherry under field conditions (Hulin, Mansfield, et al., 2018). To extend the range of sweet cherry cultivars screened for differences in resistance, detached shoot assays were conducted using representative strains from the three major canker‐causing clades Psm R1, Psm R2 and Pss, as shown in Figure 1. The strain Psm R1‐P, recognized as virulent on plum but not cherry (Hulin, Mansfield, et al., 2018), was also included (see full data Figure S1). Statistical analysis revealed significant differences in length of necrosis between cultivars (*p* < 0.01, *df* = 15), between strains (*p* < 0.01, *df* = 4) and an interaction between them (*p* < 0.01, *df* = 60). Overall, cultivars showed a large degree of variability in the length of necrotic lesion produced, which meant that apparent differences in susceptibility of many cultivars were deemed not significantly different. However, cultivars such as Merton Glory and Colney showed partial resistance to all three of the major canker pathogens, with necrosis lengths significantly lower than in the most susceptible varieties such as Van and Roundel. We previously reported that the cultivar Merton Glory exhibited partial resistance to bacterial canker (Hulin, Mansfield, et al., 2018). All cultivars showed very limited susceptibility to Psm R1‐P, the strain virulent on plum but less virulent on cherry (Figure S1).

**FIGURE 2:**
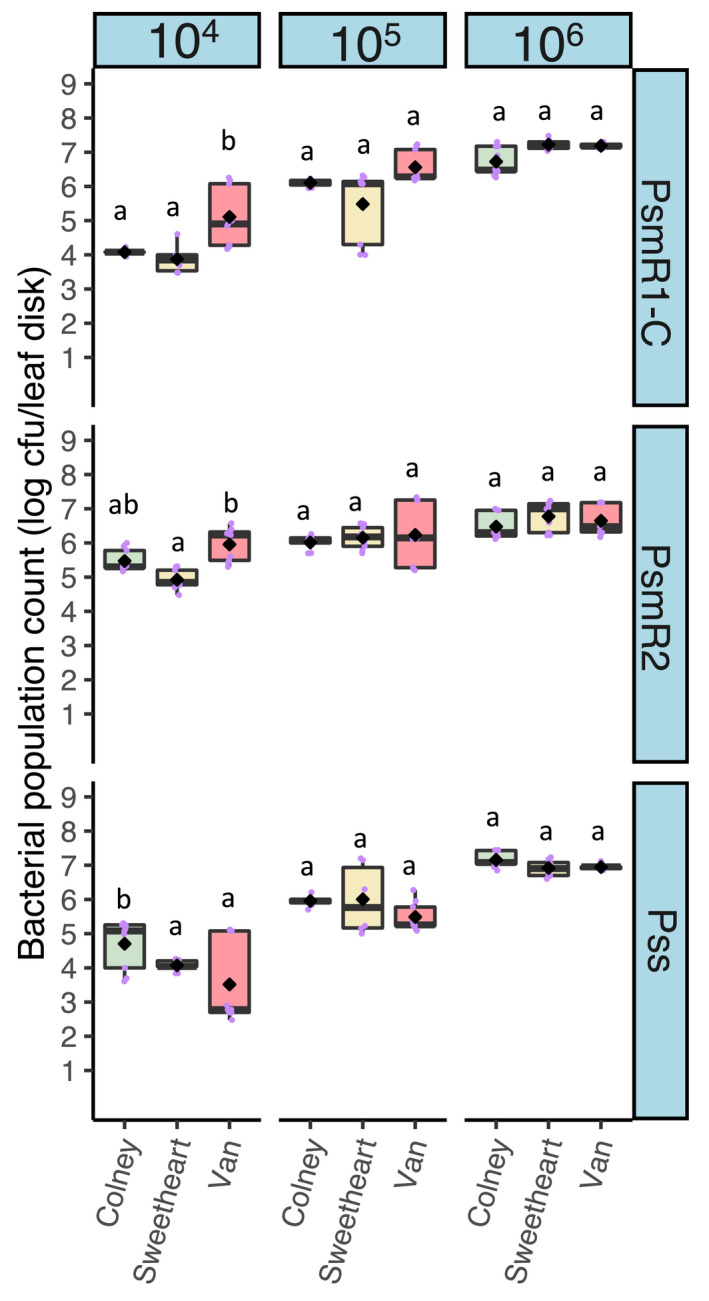
Bacterial population counts for three cherry pathogens, *Pseudomonas syringae* pv. *morsprunorum* race 1‐C (Psm R1‐C), *P*. *syringae* pv. *morsprunorum* race *2* (Psm R2) and *P*. *syringae* pv. *syringae* (Pss), after their inoculation at different concentrations (2 × 10^4^, 2 × 10^5^, 2 × 10^6^ cfu/ml) into leaves of three sweet cherry cultivars. Boxplots show the day 10 population counts for cultivars that showed differential responses in the cut‐shoot assay (Figure 1). Individual data points are included and the arithmetic mean is shown with a black diamond. This experiment was performed once. There were significant differences between strains (*p* < 0.01, *df* = 2), concentrations (*p* < 0.01, *df* = 15) and an interaction between them (*p* < 0.01, *df* = 4), while cultivars were not significantly different in this analysis (*p* = 0.055, *df* = 2). Tukey HSD (*p* = 0.05, confidence level: 0.95) significance groups for the different strains at particular concentrations are shown as letters above the boxes

In an earlier study, detached leaf syringe‐infiltration assays did not reproduce the quantitative differences seen in woody tissues of cherry varieties (Hulin, Mansfield, et al., 2018). To examine further the use of leaf inoculation to differentiate varietal resistance within sweet cherry, leaves of three cultivars that had varied in their response in the shoot assays (Figure 1), ranging from partially resistant to susceptible and highly susceptible, (Colney, Sweetheart and Van, respectively), were inoculated with progressively lower bacterial concentrations than the 10^6^ cfu/ml used in earlier work. Bacterial population counts were determined after 10 days (Figure 2). There were significant differences between strains (*p* < 0.01, *df* = 2), concentrations (*p* < 0.01, *df* = 15) and an interaction between them (*p* < 0.01, *df* = 4). However, even from the lowest inoculum level, the different cultivars did not vary significantly in final bacterial populations 10 days postinoculation (*p* = 0.055, *df* = 2). The cultivar Colney, which had exhibited reduced susceptibility in the shoot assay, did not show any reduction in bacterial populations compared to Sweetheart and Van at any of the concentrations, although at the lowest, Psm R1 and Psm R2 grew to higher levels in Van than in the other cultivars. These experiments confirmed that, in these sweet cherry cultivars, leaf infiltration inoculations did not reproduce the differential susceptibility to canker scored using cut shoots.

### 3.2 Wild cherry and other *Prunus* species exhibit leaf‐based resistance to *P. syringae*

Although leaf inoculation assays did not reproduce the differential susceptibility observed in cut shoots of sweet cherry cultivars, in previous work the more tractable leaf tests did clearly demonstrate nonhost resistance to strains of *P*. *syringae* pathogenic on other plants (Hulin, Armitage, et al., 2018; Hulin, Mansfield, et al., 2018). Therefore, we examined whether any leaf‐based resistance could be found in the wider germplasm that would give levels of resistance to the cherry pathogens comparable to nonhost resistance.

**FIGURE 3:**
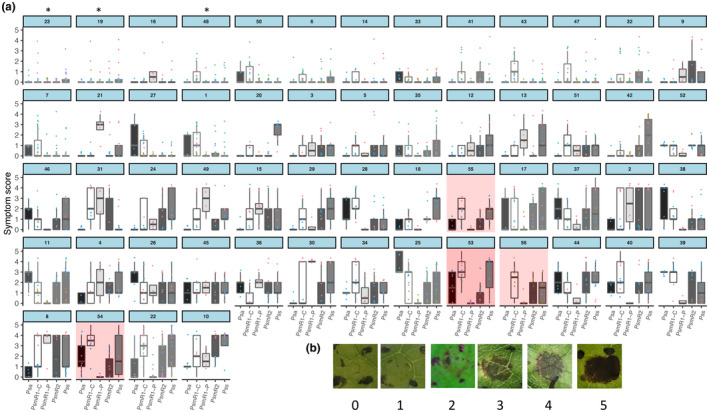
Use of leaf inoculation to screen wild cherry accessions for susceptibility to canker pathogens. (a) Boxplots of symptom scores from 52 wild cherry accessions and four sweet cherry cultivars (shaded in red) 10 days after inoculation with five *Pseudomonas syringae* strains. The strains *P*. *syringae* pv. *avii* (Psa; black), *P*. *syringae* pv. *morsprunorum* race 1‐C (Psm R1‐C; white), *P*. *syringae* pv. *morsprunorum* race 1‐P (Psm R1‐P; light grey), *P*. *syringae* pv. *morsprunorum* race 2 (Psm R2; dark grey) and *P*. *syringae* pv. *syringae* (Pss; mid grey) are coloured in different shades. Individual data points are included and coloured for each separate experiment. This experiment was performed up to five times for each strain × accession. The plots for accessions are ordered according to their resistance to infection (blue box contains accession number): 1, *Prunus avium* (P. a.) Arger Fen A; 2, Arger Fen E; 3, Barming Lane; 4, Beardown Wood; 5, Buckland Wood 8; 6, Bunny Old Wood A; 7, Bunny Old Wood B; 8, Burghley Wood; 9, Chalky Road; 10, Charger; 11, Cherryhill Copse A; 12, Chisbury Wood 1905; 13, Cobtree; 14, Coed‐Felin‐Gat; 15, Coed‐y‐Stig; 16, Deadmans Wood; 17, Dean Wood 1918; 18, Everdon Stubbs B; 19, FD1‐57‐4/122; 20, Ffynone; 21, Frydd Wood 1908; 22, Groton A; 23, Groton B; 24, Hamlet Wood C; 25, Howley Wood; 26, Lockeridge B; 27, Lowdham Lane; 28, Lower Broxford Wood A; 29, Lower Broxford Wood B; 30, Malvern Hills; 31, Marlow Common 1902; 32, Narth A; 33, Orleans‐141; 34, Pencelli Wood B; 35, Penley Wood A; 36, Postlebury B; 37, Poulton Wood A; 38, Primrose Wood; 39, Prospect Cottage; 40, Roundhill Wood; 41, Saxtens Wood B; 42, SC 311–33 (S27,S28); 43, Snarkhurst; 44, South Wood; 45, Stoke Row 1903; 46, Tank Wood; 47, Thornes Wood; 48, Thruxton Vallets; 49, Thundersley Wood; 50, Tyn‐y‐Bryn; 51, Wepre Park; 52, Wilmay Copse; and the sweet cherry cultivars: 53, Penny; 54, Sweetheart; 55, Van; 56, Colney. Ordinal regression analysis indicated that there were significant differences between cultivars (*p* < 0.01, *df* = 55) and strains (*p* < 0.01, *df* = 4). Those cultivars that showed significantly reduced symptoms across the strains compared to the least susceptible sweet cherry cultivar (55, Van) are marked with an asterisk. (b) Representative pictures of symptoms in each score category. Symptoms were scored as 0, no symptoms; 1, limited browning; 2, browning <50% of inoculated site; 3, browning >50% of inoculated site; 4, complete browning; 5, spread from site of inoculation. Infiltration sites were within the four black pen marks

Fifty‐two wild cherry accessions, and four susceptible sweet cherry accessions for comparison, were screened using young leaves from mature trees (Figure 3). In initial experiments, Psm R1, Psm R2 from cherry and plum, and Pss were used for inoculation at 2 × 10^6^ cfu/ml, and in the final screen, *P*. *syringae* pv. *avii* (Psa) was also included as this has been reported to be a pathogen of wild cherry (Ménard et al., 2003). The wild cherries exhibited a wide range of responses to the bacterial canker pathogens, from no, or very limited symptoms to complete necrosis of the inoculated region (see representative images of scores in Figure 3b). Results are presented in Figure 3a in order of the increasing severity of symptoms observed (mean overall symptom score per cultivar). Several accessions produced limited or no symptoms during this screening. In particular, the wild cherries P.a. Groton B, P. a. FD1‐57‐4/122, P. a. Deadmans Wood and P. a. Thruxton Vallets (numbered 23, 19, 16 and 48, respectively, in Figure 3a) were scored as highly resistant.

Ordinal statistical analysis confirmed that there were significant differences between accessions (*p* < 0.01, *df* = 55) and between strains (*p* < 0.01, *df* = 4). However, an interaction model could not be fitted due to complete separation of the response factor preventing model convergence (e.g., where in selected cases all scores were the same for a particular strain × cultivar combination) as discussed in Allison (2008). Nevertheless, in some genotypes there were clear differential reactions to the pathogenic strains (listed in Table S3). For example, genotypes 15 (Coed‐y‐Stig) and 25 (Howley Wood) showed resistance to Psa and Psm R1‐P, respectively, but were susceptible to other strains. Sweet cherry cultivars were resistant to the plum strain Psm R1‐P (graphs shaded in red in Figure 3a), but several wild cherries were susceptible, for example, 31 (Marlow Common 1902) and 21 (Frydd Wood 1908), the latter recording very little symptom development with the other strains. Another pattern to emerge was lesion formation following inoculation with Psa and Psm R1‐C from cherry, but resistance to other strains as recorded in accessions 1, Arger Fen A; 7, Bunny Old Wood B; 27, Lowdham Lane and 50, Tyn‐y‐Bryn. The statistical analysis indicated that lesions on accessions 23, P.a. Groton B; 19, P. a. FD1‐57‐4/122 and 48, P. a. Thruxton Vallets were significantly reduced compared to sweet cherry controls. Other possibly resistant accessions, such as 16, P. a. Deadmans Wood, were not deemed significantly different (based on Tukey post hoc groupings), which may have been due to reduced data for this accession.

**FIGURE 4:**
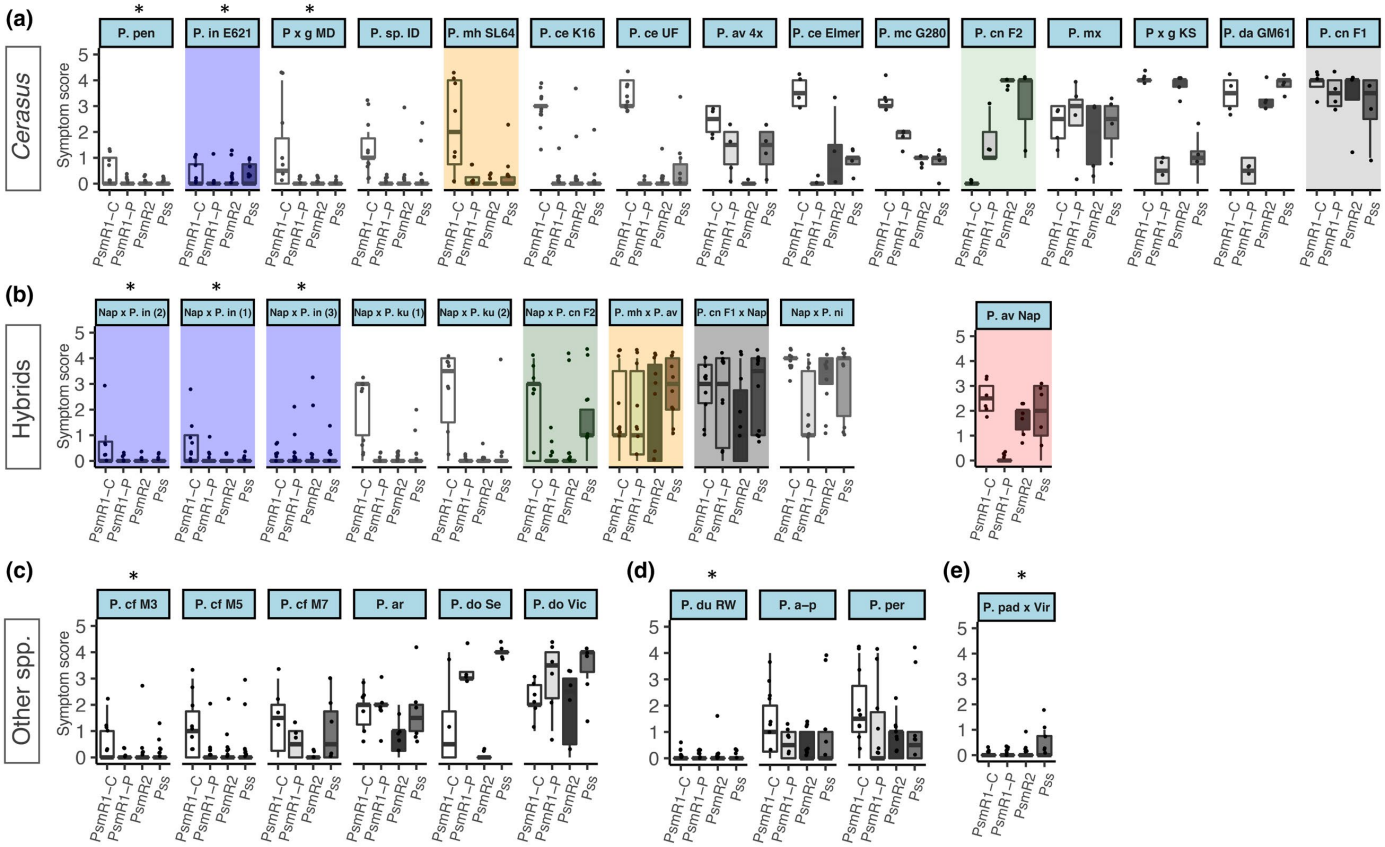
Leaf inoculation‐based screen of a range of *Prunu*s species and hybrids (see Table 1 for full descriptions) for susceptibility to cherry canker pathogens. The boxplots show symptom scores 10 days after inoculation with four *Pseudomonas syringae* strains. The strains *P*. *syringae* pv. *morsprunorum* race 1‐C (Psm R1‐C), *P*. *syringae* pv. *morsprunorum* race 1‐P (Psm R1‐P), *P*. *syringae* pv. *morsprunorum* race 2 (Psm R2) and *P*. *syringae* pv. *syringae* (Pss) are coloured in shades of grey. Individual data points are included.. This experiment was performed up to two times for each strain × accession. (a) *Prunus* subgenus *Cerasus*, (b) *Prunus avium* hybrids, (c) *Prunus* subgenus *Prunus*, (d) *Prunus* subgenus *Amygdalus*, (e) *Prunus* subgenus *Padus*. Where the hybrids in (b) were also screened, the plot is shaded to show this (e.g., *P*. *incisa* E621 in (a) is the parent of three hybrids coloured in blue). *P*. *avium* 'Napoleon' (highlighted in red) was a parent of most hybrids (see Table 1 for more details and abbreviations). Ordinal regression analysis indicated that there were significant differences between cultivars (*p* < 0.01, *df* = 34) and strains (*p* < 0.01, *df* = 3). The accessions that showed significantly reduced symptoms across the strains compared to cherry cultivar Napoleon (P. av Nap) are marked with an asterisk. Symptoms were scored as 0, no symptoms; 1, limited browning; 2, browning <50% of inoculated site; 3, browning >50% of inoculated site; 4, complete browning; 5, spread from site of inoculation.

Subsequently, screening by leaf inoculation was extended to a range of other *Prunus* species using Psm R1‐C, Psm R1‐P, Psm R2 and Pss (Figure 4), which are the main pathogens of cherry. Species tested included members of the subgenus *Cerasus* (Figure 4a), sweet cherry interspecific hybrids with other *Cerasus* species (Figure 4b), subgenus *Prunus* (Figure 4c), subgenus *Amygdalus* (Figure 4d) and subgenus *Padus* (Figure 4e). Statistical analysis again indicated that there were significant differences between accessions (*p* < 0.01, *df* = 34) and between strains (*p* < 0.01, *df* = 3). Those with significantly less symptom development overall, compared to sweet cherry (cv. Napoleon, as this was a parent of most of the interspecific hybrids) are marked by asterisks in Figure 4. Accessions of *P*. *dulcis*, *P*. *cerasifera*, *P*. *padus*, *P*. *pensylvanica*, *Prunus* × *gondouinii* and *P*. *incisa* all exhibited very limited to no symptom development when inoculated with the major cherry pathogens. Interspecific hybrids of sweet cherry with other species within the *Cerasus* subgenus (Figure 4b) included three progeny from a *P*. *incisa* × *P*. *avium* sweet cherry cross, and all failed to develop significant lesions.

**FIGURE 5:**
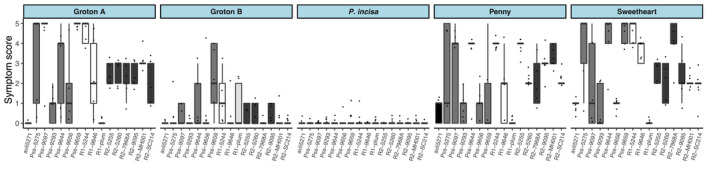
Screening of accessions of wild cherry (Groton A and B), ornamental cherry (*Prunus incisa*) and sweet cherry (Penny, Sweetheart) with 16 strains of the cherry canker pathogen *Pseudomonas syringae*. The boxplots show symptom scores 10 days after inoculation. The strains are coloured in shades of grey by clade: *P*. *syringae* pv. *avii* (Psa), *P*. *syringae* pv. *morsprunorum* race 1‐C (Psm R1‐C), *P*. *syringae* pv. *morsprunorum* race 1‐P (Psm R1‐P), *P*. *syringae* pv. *morsprunorum* race 2 (Psm R2) and *P*. *syringae* pv. *syringae* (Pss). Individual data points are included and the experiment was performed only once. Ordinal analysis confirmed differences between cultivars (*p* < 0.01, *df* = 4). Symptoms were scored as 0, no symptoms; 1, limited browning; 2, browning <50% of inoculated site; 3, browning >50% of inoculated site; 4, complete browning; 5, spread from site of inoculation

Leaves of several accessions of wild cherry and other *Prunus* species developed limited symptoms after inoculation with the major cherry pathogens. To determine if this resistance operated against a wider range of isolates from each pathogenic clade, two of the most resistant accessions (wild cherry Groton B and ornamental species *P. incisa*), as well as susceptible sweet (Penny and Sweetheart) and wild (Groton A) cherry cultivars for comparison, were screened with 16 previously characterized *P*. *syringae* strains pathogenic on cherry and plum (Figure 5). The wild cherry Groton B generally recorded low levels of symptom development, but a tree from the same woodland, Groton A, was highly susceptible and comparable to the sweet cherry varieties (Figure 3). This test with further strains confirmed that Groton B exhibited resistance, although some strains of Pss were able to cause lesions. Inoculation with each of the 16 strains tested failed to cause symptoms in the ornamental species *P*. *incisa*. Statistical analysis confirmed differences between cultivars (*p* < 0.01, *df* = 4), with Groton B and *P*. *incisa* recording significantly lower symptom scores with all pathogenic strains.

### 3.3 The more resistant varieties of wild and ornamental cherry support lower in planta bacterial multiplication

**FIGURE 6:**
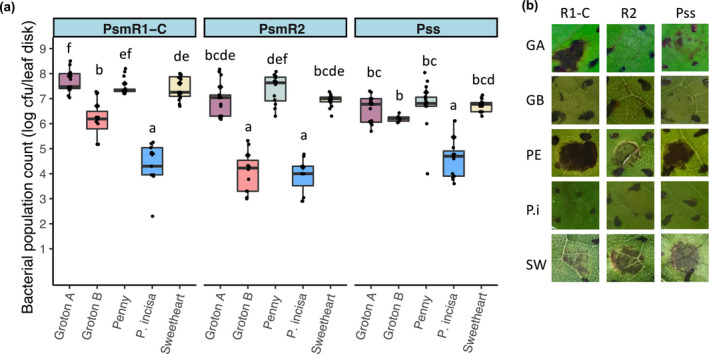
Bacterial population counts of cherry pathogens *Pseudomonas syringae* pv. *morsprunorum* race 1‐C (Psm R1‐C), *P*. *syringae* pv. *morsprunorum* race 2 (Psm R2) and *P*. *syringae* pv. *syringae* (Pss) inoculated into leaves of sweet cherry (Penny, Sweetheart), wild cherry (Groton A and Groton B) and ornamental cherry (*Prunus incisa*). (a) Boxplots show the day 10 population counts for each strain on each cultivar after inoculation with 2 × 10^6^ cfu/ml of each strain. Individual data points are included and the arithmetic mean is shown with a black diamond. This experiment was performed once. Analysis of variance revealed there were significant differences between strains (*p* < 0.01, *df* = 2) and cultivars (*p* < 0.01, *df* = 4) as well as an interaction between them (*p* < 0.01, *df* = 8). Tukey HSD (*p* = 0.05, confidence level: 0.95) significance groups for the whole data set comparison are labelled. (b) Representative pictures of disease symptoms for each strain × cultivar combination (images taken during initial screens documented in Figures 3 and 4); infiltration sites were within the four black pen marks. Note the lack of macroscopic lesions in *P*. *incisa*

The wild cherry Groton B and ornamental species *P*. *incisa* had shown a high level of resistance. To establish if bacterial multiplication was reduced within the leaves of these cultivars, populations were counted 10 days after inoculation (Figure 6a). Two susceptible sweet cherries and a susceptible wild cherry, Groton A, from the same forest as Groton B, were included for comparison. Representative images of symptoms taken during initial screens of these accessions are displayed in Figure 6b. Statistical analysis revealed that there were significant differences between strains (*p* < 0.01, *df* = 2) and accessions (*p* < 0.01, *df* = 4) as well as an interaction between them (*p* < 0.01, *df* = 8). The more resistant genotypes, Groton B and *P*. *incisa*, supported lower bacterial populations of both Psm R1 and Psm R2 10 days postinoculation, and showed limited or no symptom development compared to susceptible cultivars. Multiplication of Pss was not significantly lower in Groton B than in the susceptible sweet cherry cultivars (Penny and Sweetheart) in this experiment, but *P*. *incisa* again proved to be resistant.

### 3.4 Relationship between resistance response and bacterial inoculum dose

**FIGURE 7:**
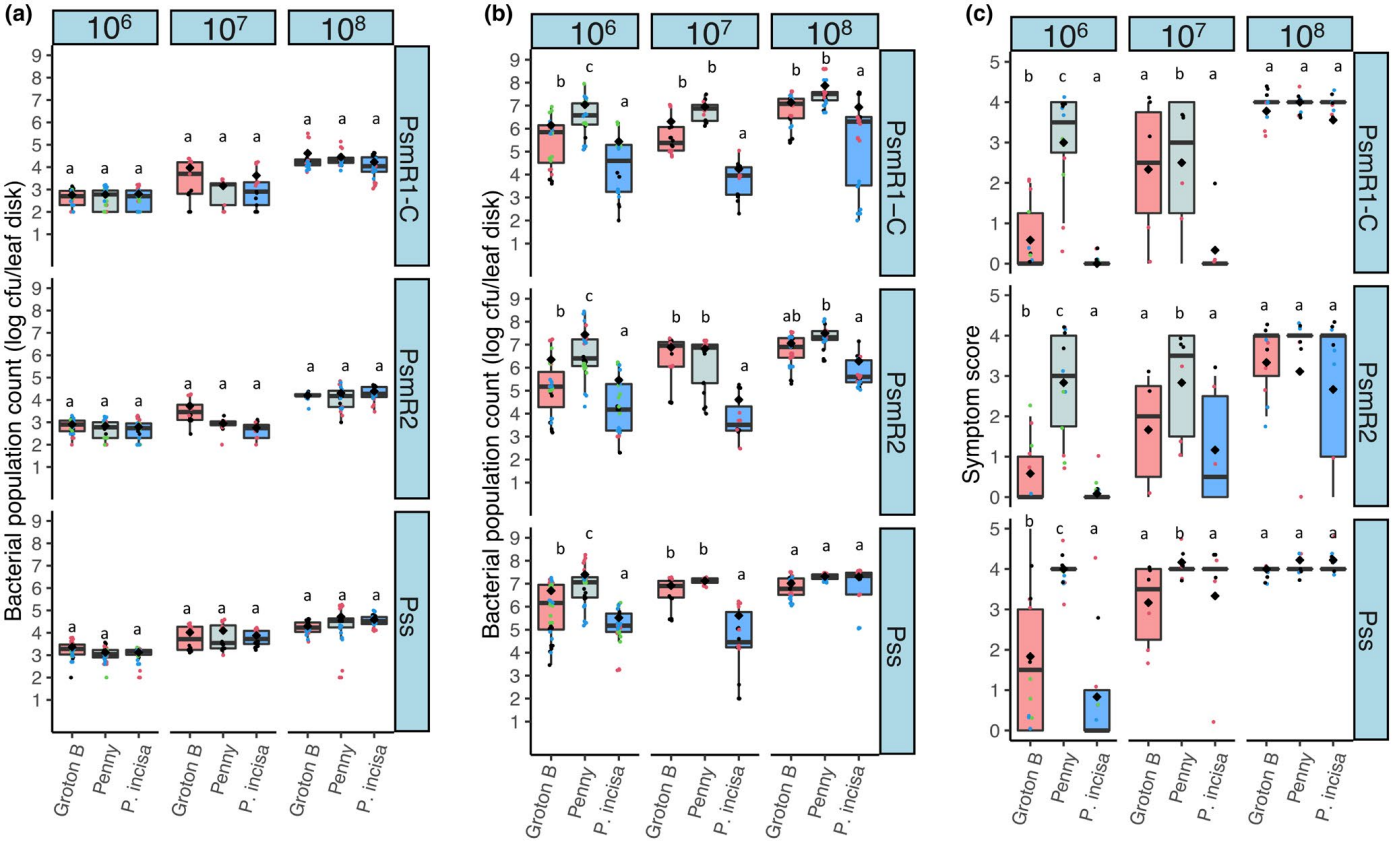
Bacterial population counts of cherry pathogens, *Pseudomonas syringae* pv. *morsprunorum* race 1‐C (Psm R1‐C), *P*. *syringae* pv. *morsprunorum* race 2 (Psm R2) and *P*. *syringae* pv. *syringae* (Pss), inoculated into leaves of wild cherry (Groton B), sweet cherry (Penny) and ornamental cherry (*Prunus incisa*) at different inoculum concentrations. (a) Boxplots show the day 0 population counts for cultivars. Individual data points are included and coloured for each separate experiment and the arithmetic mean is shown with a black diamond. This experiment was repeated up to four times per cultivar × strain combination. Analysis of variance (ANOVA) revealed a significant difference between strains (*p* < 0.01, *df* = 2), concentrations (*p* < 0.01, *df* = 2) and an interaction between them (*p* < 0.01, *df* = 4). There was no significant difference in bacterial populations between cultivars (*p* = 0.32, *df* = 2). Tukey HSD (*p* = 0.05, confidence level: 0.95) significance groups for the different strains at particular concentrations are presented. (b) Boxplots show the day 10 population counts for cultivars. The layout is the same as in (a). ANOVA revealed a significant difference between strains (*p* < 0.01, *df* = 2), cultivars (*p* < 0.01, *df* = 2), concentrations (*p* < 0.01, *df* = 2) and a cultivar × strain interaction (*p* < 0.01, *df* = 4), cultivar × concentration interaction (*p* < 0.01, *df* = 4) and strain × concentration interaction (*p* = 0.03, *df* = 4). (c) Symptom scores at day 10, scored as 0, no symptoms; 1, limited browning; 2, browning <50% of inoculated site; 3, browning >50% of inoculated site; 4, complete browning; 5, spread from site of inoculation. Data are presented as in (a) and (b). Ordinal analysis revealed a significant difference between strains (*p* < 0.01, *df* = 2), concentrations (*p* < 0.01, *df* = 2), cultivars (*p* < 0.01, *df* = 2) and a cultivar × concentration interaction (*p* < 0.01, *df* = 4)

To see if the observed resistance in certain accessions was robust to increasing concentrations of bacterial inoculum, Groton B, *P*. *incisa* and the susceptible cultivar Penny were inoculated using increasing doses ranging from 2 × 10^6^ cfu/ml to 2 × 10^8^ cfu/ml (Figure 7). At day 0 (Figure 7a), there was no significant difference between bacterial numbers in accessions (*p* = 0.32, *df* = 2). At day 10, the wild cherry Groton B supported high bacterial populations of all pathogens when inoculated at 2 × 10^7^ cfu/ml and 2 × 10^8^ cfu/ml, with resistance only apparent at the lower inoculum concentration (Figure 7b). By contrast, the ornamental species *P*. *incisa* recorded significantly reduced bacterial populations even when inoculated at 2 × 10^8^ cfu/ml with Psm R1 and Psm R2, although Pss appeared to overcome any resistance using the highest inoculum concentration. Symptom scoring in these experiments revealed that at the lower concentration (2 × 10^6^ cfu/ml) Groton B and *P*. *incisa* recorded very limited symptom formation after 10 days (Figure 7c), confirming the results presented in Figures 3 and 4. By contrast, at the higher inoculum concentrations, symptoms were more apparent and similar to those observed in sweet cherry cv. Penny, particularly for the more virulent Pss.

**FIGURE 8:**
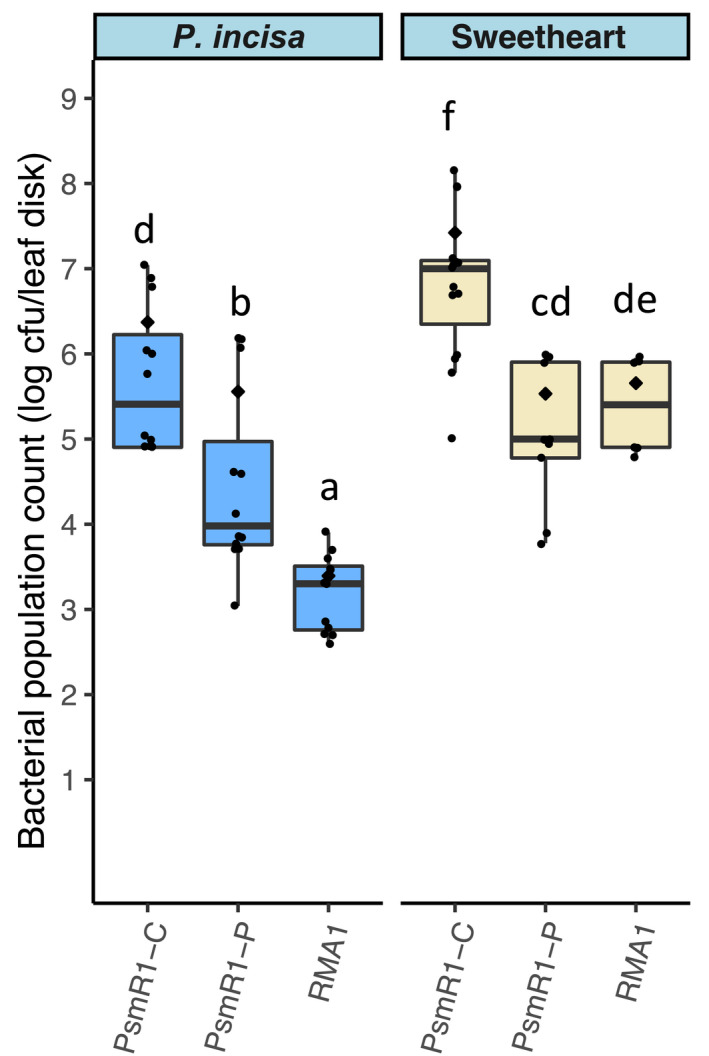
Bacterial populations following inoculation at 2 × 10^8^ cfu/ml into leaves of *Prunus incisa* and sweet cherry cv. Sweetheart of a cherry pathogen *Pseudomonas syringae* pv. *morsprunorum* race 1‐C (Psm R1‐C) and two strains of *P*. *syringae* originating from different plants (from plum, *P*. *syringae* pv. *morsprunorum* race 1‐P [Psm R1‐P] and from *Aquilegia vulgaris* [RMA1]) that are nonpathogenic to cherry. Boxplots show the day 4 population counts for cultivars. Individual data points are included and the arithmetic mean is shown with a black diamond. This experiment was performed once. Analysis of variance revealed a significant difference between strains (*p* < 0.01, *df* = 2), cultivars (*p* < 0.01, *df* = 1) and an interaction between them (*p* < 0.01, *df* = 2). Tukey HSD (*p* = 0.05, confidence level: 0.95) significance groups comparing all cultivar × strain combinations are presented (letters a–f)

The restriction of bacterial populations in *P*. *incisa*, particularly towards Psm R1 and Psm R2 at higher inoculum concentrations, was similar to a nonhost resistance response as seen previously in cherry towards plum and *Aquilegia* pathogens (Hulin, Mansfield, et al., 2018). To examine if the multiplication of the sweet cherry pathogen Psm R1‐C was similar to nonpathogens of cherry in *P*. *incisa*, several strains were inoculated at the highest inoculum concentration (2 × 10^8^ cfu/ml) on *P*. *incisa* and compared with a susceptible cherry, 4 days after infiltration (Figure 8). The nonpathogens Psm R1‐P from plum and RMA1 (a pathogen of *Aquilegia*) reached levels of 1 × 10^5^ to 1 × 10^6^ cfu/leaf disk in cherry cv. Sweetheart, while the pathogenic strain Psm R1‐C grew 10 times higher. Psm R1‐C did not grow as well in *P*. *incisa* where it reached levels of 1 × 10^5^ to 1 × 10^6^ cfu/leaf disk. However, the nonpathogens of cherry multiplied even less in *P*. *incisa* than they did in the sweet cherry. These results indicated that Psm R1‐C may be more adapted to *P*. *incisa* than strains originating from unrelated plant hosts even though the ornamental cherry species still appears to have significant resistance.

**FIGURE 9:**
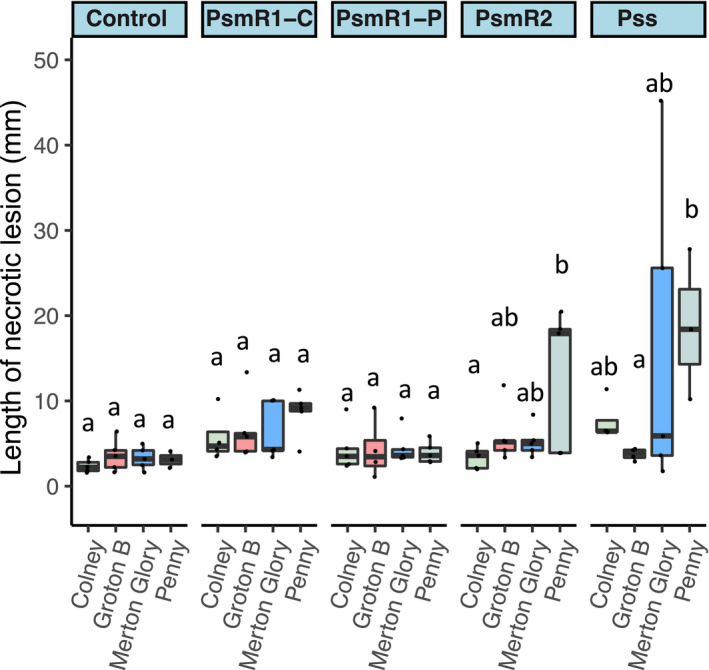
Susceptibility of sweet (Colney, Merton Glory and Penny) and wild cherry (Groton B) cultivars to *Pseudomonas syringae* infection using cut shoots. Boxplots show length of necrotic lesions in cut shoots 6 weeks after inoculation with the cherry pathogens *Pseudomonas syringae* pv. *morsprunorum* race 1‐C (Psm R1‐C), *P*. *syringae* pv. *morsprunorum* race 2 (Psm R2), *P*. *syringae* pv. *syringae* (Pss) or the plum pathogen *P*. *syringae* pv. *morsprunorum* race 1‐P (Psm R1‐P). Individual data points are included. This experiment was performed once. Analysis of variance revealed a significant interaction between strains (*p* < 0.01, *df* = 4) and cultivars (*p* = 0.01, *df* = 3). Note the resistance of Groton B to all strains

Finally, to confirm if the resistance response of Groton B and *P*. *incisa* seen in leaves was reflected in woody tissue, a cut‐shoot assay was performed (Figure 9). Unfortunately, the *P*. *incisa* shoots were not amenable to this assay and dried out, probably due to their thinness. However, the assay confirmed that Groton B, like the more resistant sweet cherry cultivars Merton Glory and Colney, showed much reduced necrosis compared to the susceptible sweet cherry Penny.

## 4 DISCUSSION

The development of rapid laboratory‐based tests to allow screening for resistance in trees is a major challenge that underpins the rapid development of new cultivars that resist pests and diseases. Hulin, Mansfield, et al. (2018) addressed this issue in relation to cherry canker and found that cut‐shoot assays most closely reflected canker disease development in whole‐tree tests in the field. Although the more tractable leaf inoculation failed to differentiate sweet cherry cultivar resistance levels, it did allow clear differentiation between the canker pathogens and pathogens of other plants. Nonhost resistance was well defined in leaves and reflected the failure of the nonpathogens to cause symptoms in woody tissues. In the present study, we describe further analysis of partial resistance in sweet cherry cultivars and use a leaf infection‐based screen of wild cherry and related *Prunus* spp. to identify potential new sources of resistance to all clades of *P*. *syringae* that cause cherry canker. Arguably, assays on woody tissues, such as shoots or whole trees, are required to fully determine bacterial canker resistance in breeding programmes. However, the use of nonwoody material for screening provided a rapid way to search for strong resistance phenotypes and has been used in other studies, including detached leaves (Mgbechi‐Ezeri et al., 2017) and micropropagated plantlets (Vicente & Roberts, 2003).

In our first experiments, we inoculated a range of sweet cherry cultivars with *P*. *syringae*, and detected variation in susceptibility to Psm R1, Psm R2 and Pss in the woody tissue (cut shoots) but not in leaf tissue, even at low inoculum concentrations. This suggested that perhaps leaf assays are not sensitive enough to pick up small differences in cultivar susceptibility, or perhaps tissue‐specific differences in immune responses may occur. Further studies using less mechanical methods, that do not bypass surface‐based immunity, such as spray or dip inoculations of leaves, might reveal subtle differences between cultivars (Liu et al., 2015). We do not know what mechanisms of partial resistance are operating in woody shoots of the less susceptible cultivars such as Colney and Merton Glory. The differences in lesion formation observed could be due to the physical structure of the woody tissues rather than some differential biochemical defence response. The more susceptible varieties might have larger intercellular spaces between cambial tissues that allow more rapid, unrestricted bacterial colonization from the cut end of the shoot. Such a tissue‐based difference would explain the lack of expression of resistance in leaves where a dynamic, cellular response may be the key to prevention of colonization. These hypotheses remain to be tested. Although woody tissues are, arguably, the main sites of infection by *P*. *syringae* causing canker disease, other tissues such as leaves and blossom can be colonized and harbour the pathogen (Crosse, 1966) and resistance in these tissues is of use for breeding programmes.

Although, the responses of sweet cherry cultivars tested could not be differentiated on leaves, we reasoned that relatives of sweet cherry might exhibit resistance in nonwoody tissues as seen in previous work (Vicente & Roberts, 2003). A large screen of diverse wild cherry revealed several accessions, notably Groton B and FD1‐57‐4/122, that exhibited resistance to strains from all the canker‐producing *P*. *syringae* clades. These data support previous observations during projects focused on wild cherry. Groton B was identified as being significantly more resistant in cut‐shoot tests in 1996 and 1998 at EMR (K. Russell, unpublished data). Similarly, FD1‐57‐4/122 is a seedling selection bred at East Malling from a wild mazzard seedling F1/3a, originally introduced in 1914. F1/3a was shown to have canker resistance when screened in a clonal rootstock breeding programme at East Malling (Garrett, 1979). A sibling of FD1‐57‐4/122, FD1‐57‐4/166, was also found to be more resistant in plantlet assays (Vicente & Roberts, 2003).

Differential symptom development in some accessions also suggests the existence of a pattern of resistance and susceptibility, as observed in examples of race‐ and cultivar‐specific resistance in other plant–bacterium interactions, for example in bean halo blight disease (Arnold et al., 2011). Differential reactions observed are listed in Table S3, but no simple model based on the presence of *R* genes matching each clade could be fitted to the data. The reactions observed to the plum strain Psm R1‐P are of particular interest. Resistance to Psm R1‐P in sweet cherry could be due to resistance triggered by the intracellular detection of pathogen effectors such as HopAB1 by the plant immune system. Genomic analysis revealed the *hopAB1* effector gene is present in this strain but not its relatives that are pathogenic on cherry (Hulin, Armitage, et al., 2018; Hulin, Mansfield, et al., 2018). Several wild *P*. *avium* accessions were susceptible to infection by the plum strain, developing distinct lesions, and presumably these accessions could lack a receptor recognizing HopAB1, such as Pto in tomato species (Chien et al., 2013). The role of HopAB1 as an inducer of effector‐triggered immunity and/or a virulence determinant should be tested by genetic dissection through deletion of *hopAB1* from Psm R1‐P.

The study was extended to other *Prunus* species and sweet cherry hybrids. In particular, some *Prunus* species also displayed resistance to the major pathogen strains, and the Fuji cherry accession *P*. *incisa* proved resistant to all 16 canker pathogens tested. The resistance suggested by lack of symptom development in wild cherry and related *Prunus* spp. was confirmed through analysis of bacterial multiplication in leaves. Bacterial populations reached in *P*. *incisa* were lower than those recorded in the selected wild cherry accession Groton B. The dynamics of population growth in *P*. *incisa* were similar to those recorded for nonpathogens in sweet cherry. The similarly reduced multiplication of the nonhost plum and *Aquilegia* pathogens in *P*. *incisa* compared with sweet cherry indicates that there may be a more rapid deployment of resistance, perhaps mediated through an enhanced level of cell surface‐based immunity and/or effector‐triggered intracellular responses. Whatever the biochemical nature of resistance, the lack of symptoms found in the hybrids between *P*. *incisa* and the sweet cherry cv. Napoleon after challenge with the major pathogens suggests that the resistance from *P*. *incisa* is probably inherited as a dominant trait.

The resistant wild cherry and *Prunus* accessions selected, Groton B and *P*. *incisa*, respectively, have now been incorporated into breeding programmes to introgress resistance into commercial sweet cherry genotypes and generate more resistant varieties for growers. Progeny of Groton B have also been selected for wild cherry‐breeding programmes to improve canker resistance in the forestry industry (K. Russell, unpublished data). Such work can take up to 15 years. The routine testing of progeny performance against the main canker pathogens during these projects and future genetic research will provide further insights into the genetic controls underlying the outcome of the *Prunus–P*. *syringae* interaction.

## CONFLICT OF INTEREST

The authors declare no conflict of interest.

## Supporting information

Fig S1

Table S1

Table S2

Table S3

## Data Availability

The data that support the findings of this study are available from the corresponding author upon reasonable request.
