# Supplementary figures and images for: Identifying resistance in wild and ornamental cherry towards bacterial canker caused by Pseudomonas syringae
Source: Plant Pathol. 2021 Dec 21;71(4):949–65. doi: 10.1111/ppa.13513 (PMC9305585; doi:10.1111/ppa.13513)

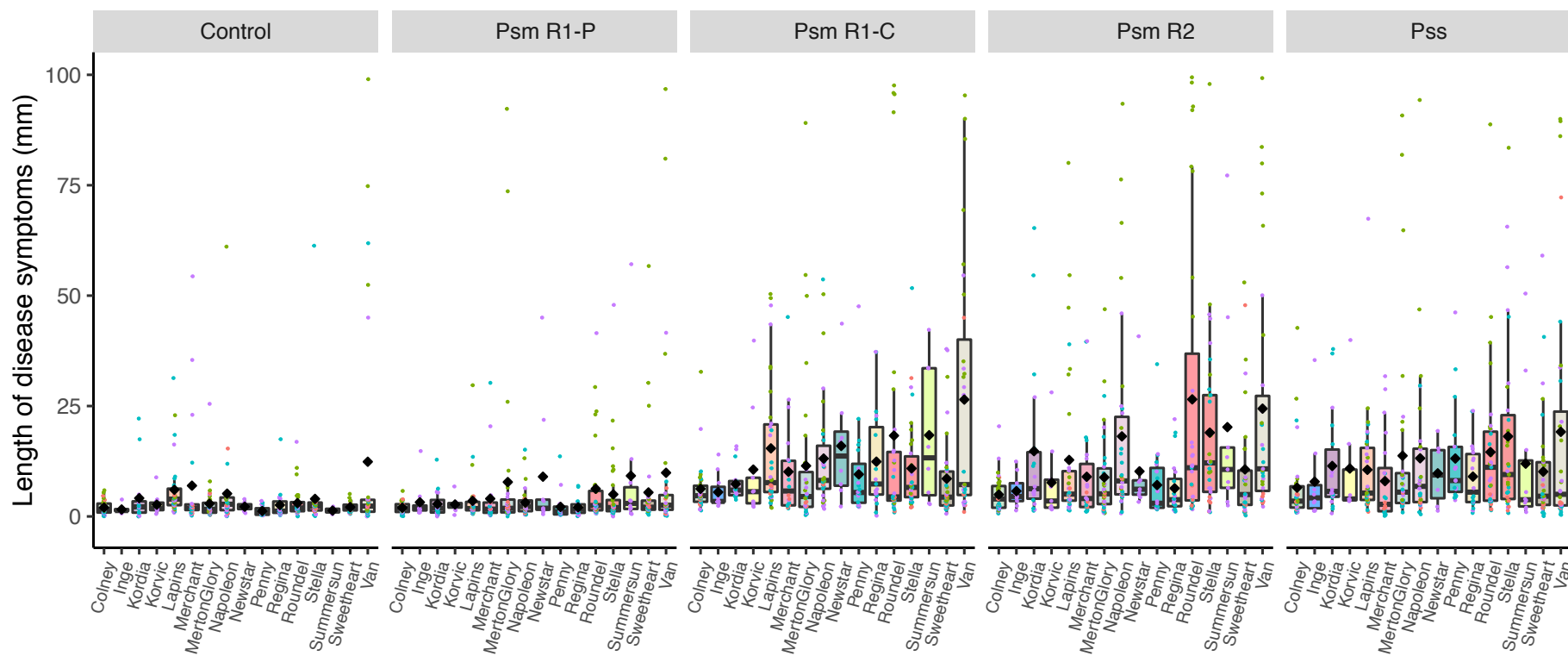

Supplement: Supplementary file 1 — Fig S1 [file PPA-71-949-s004.pdf]
