# Supplementary material for: Identifying resistance in wild and ornamental cherry towards bacterial canker caused by Pseudomonas syringae
Source: Plant Pathol. 2021 Dec 21;71(4):949–65. doi: 10.1111/ppa.13513 (PMC9305585; doi:10.1111/ppa.13513)
Supplement: Supplementary file 2 — Table S1 [file PPA-71-949-s003.docx]

Table S1. Origins of *Prunus* *avium* wild cherry accessions screened in this study. ^a^: Bred at NIAB EMR in Kent. Blank fields are unknown. Grid ref refers to ordnance survey grid reference.

| Accession | No. | County | Grid ref | Longitude | Latitude |
| --- | --- | --- | --- | --- | --- |
| P. a. Arger Fen A | 1 | Suffolk | TL934355 | 51.98428 | 0.81496 |
| P. a. Arger Fen E | 2 | Suffolk | TL933355 | 51.98432 | 0.813506 |
| P. a. Barming Lane | 3 | Kent | TQ716550 | 51.26832 | 0.458425 |
| P. a. Beardown Wood | 4 | Devon | SS780073 | 50.85231 | -3.73452 |
| P. a. Buckland Wood 8 | 5 | Buckinghamshire | SP909078 | 51.76152 | -0.6843 |
| P. a. Bunny Old Wood A | 6 | Nottinghamshire | SK581282 | 52.84818 | -1.13874 |
| P. a. Bunny Old Wood B | 7 | Nottinghamshire | SK583283 | 52.84905 | -1.13576 |
| P. a. Burghley Wood | 8 | Lincolnshire | TF022048 | 52.63134 | -0.4914 |
| P. a. Chalky Road | 9 | Kent |  |  |  |
| P. a. Charger ^a^ | 10 | Kent | TQ710568 | 51.28467 | 0.450693 |
| P. a. Cherryhill Copse A | 11 | Hampshire | SU641128 | 50.91112 | -1.08963 |
| P. a. Chisbury Wood 1905 | 12 | Wiltshire | SU269652 | 51.38521 | -1.61483 |
| P. a. Cobtree | 13 | Kent | TQ744588 | 51.30161 | 0.500375 |
| P. a. Coed-Felin-Gat | 14 | Carmarthenshire | SN525188 | 51.84826 | -4.14279 |
| P. a. Coed-y-Stig | 15 | Denbighshire | SJ087611 | 53.1392 | -3.36629 |
| P. a. Deadmans Wood | 16 | Kent | TQ723568 | 51.28428 | 0.469316 |
| P. a. Dean Wood 1918 | 17 | Buckinghamshire | SU972909 | 51.60855 | -0.59775 |
| P. a. Everdon Stubbs B | 18 | Northamptonshire | SP606564 | 52.20251 | -1.1147 |
| P. a. FD1-57-4/122 ^a^ | 19 | Kent | TQ710568 | 51.28467 | 0.450693 |
| P. a. Ffynone | 20 | Pembrokeshire | SN239385 | 52.0169 | -4.56768 |
| P. a. Frydd Wood 1908 | 21 | Powys | SO075901 | 52.50093 | -3.36409 |
| P. a. Groton A | 22 | Suffolk | TL976432 | 52.05195 | 0.88048 |
| P. a. Groton B | 23 | Suffolk | TL976432 | 52.05195 | 0.88048 |
| P. a. Hamlet Wood C | 24 | Kent | TQ745526 | 51.24588 | 0.498783 |
| P. a. Howley Wood | 25 | Gloucestershire | SO666210 | 51.88655 | -2.48669 |
| P. a. Lockeridge B | 26 | Devon | SX438665 | 50.47745 | -4.2028 |
| P. a. Lowdham Lane | 27 | Nottinghamshire | SK646477 | 53.0227 | -1.03837 |
| P. a. Lower Broxford Wood A | 28 | Devon | SS847032 | 50.81683 | -3.63809 |
| P. a. Lower Broxford Wood B | 29 | Devon | SS844031 | 50.81588 | -3.64232 |
| P. a. Malvern Hills | 30 | Worcestershire | SO771430 | 52.08487 | -2.33561 |
| P. a. Marlow Common 1902 | 31 | Buckinghamshire | SU827864 | 51.57042 | -0.80815 |
| P. a. Narth A | 32 | Monmouthshire | SO528061 | 51.75159 | -2.68515 |
| P. a. Orleans-141 | 33 | Pas de Calais |  |  |  |
| P. a. Pencelli Wood B | 34 | Powys | SO085252 | 51.91778 | -3.3318 |
| P. a. Penley Wood A | 35 | Wrexham | SJ419407 | 52.96051 | -2.86638 |
| P. a. Postlebury B | 36 | Somerset | ST741433 | 51.18833 | -2.37198 |
| P. a. Poulton Wood A | 37 | Kent | TR058365 | 51.09088 | 0.937445 |
| P. a. Primrose Wood | 38 | East Sussex | TQ545325 | 51.07104 | 0.20385 |
| P. a. Prospect Cottage | 39 | Gloucestershire | SO531040 | 51.73274 | -2.68052 |
| P. a. Roundhill Wood | 40 | Hertfordshire | SP939086 | 51.76821 | -0.64063 |
| P. a. Saxtens Wood B | 41 | Kent | TQ584647 | 51.35929 | 0.27368 |
| P. a. SC 311-33 (S27,S28) | 42 | Kent | TQ588651 | 51.36277 | 0.279599 |
| P. a. Snarkhurst | 43 | Kent | TQ825556 | 51.27033 | 0.614808 |
| P. a. South Wood | 44 | Surrey | TQ077345 | 51.0997 | -0.46323 |
| P. a. Stoke Row 1903 | 45 | Oxfordshire | SU666849 | 51.55906 | -1.04069 |
| P. a. Tank Wood | 46 | Kent | TQ906326 | 51.0611 | 0.718607 |
| P. a. Thornes Wood | 47 | Devon | SS985105 | 50.88504 | -3.44429 |
| P. a. Thruxton Vallets | 48 | Hertfordshire | SO439335 | 51.9971 | -2.81852 |
| P. a. Thundersley Wood | 49 | Essex | TQ785881 | 51.56353 | 0.573882 |
| P. a. Tyn-y-Bryn | 50 | Powys | SJ053062 | 52.64524 | -3.40109 |
| P. a. Wepre Park | 51 | Flintshire | SJ297682 | 53.2062 | -3.05399 |
| P. a. Wilmay Copse | 52 | Kent | TQ579655 | 51.36662 | 0.26686 |
