# Supplementary material for: Identifying resistance in wild and ornamental cherry towards bacterial canker caused by Pseudomonas syringae
Source: Plant Pathol. 2021 Dec 21;71(4):949–65. doi: 10.1111/ppa.13513 (PMC9305585; doi:10.1111/ppa.13513)
Supplement: Supplementary file 3 — Table S2 [file PPA-71-949-s002.docx]

Table S2: Strains of *Pseudomonas syringae* used in this study with host of origin and original isolator. Experiment lists which experiments each strain was used for; a: sweet cherry cut-shoot (Fig. 1, Fig. S1), b: sweet cherry leaf populations with different inoculum concentrations (Fig. 2), c: wild cherry leaf symptom screen (Fig. 3), d: other *Prunus* species leaf screen (Fig. 4), e: Selected accessions large leaf symptom screen with sixteen bacterial strains (Fig. 5), f: Selected accessions leaf population counts (Fig. 6), g: Selected accession leaf population counts at different inoculum concentrations (Fig. 7), h: Leaf population count with non-host *P. syringae* strains (Fig. 8), i: Cut shoot inoculation with selected accessions (Fig. 9). * Strains not pathogenic on sweet cherry in previous study (Hulin et al. 2018a), all other strains are pathogenic on cherry.

| Strain | Clade | Plant host | Isolator | Experiment |
| --- | --- | --- | --- | --- |
| R1-5244 | *P. syringae* pv *morsprunorum* R1 | *Prunus avium* | Garrett, 1990 | abcdefghi |
| R1-plum/R1-5300* | *P. syringae* pv *morsprunorum* R1 | *Prunus domestica* | Garrett, 1990 | acdehi |
| R1-9646 | *P. syringae* pv *morsprunorum* R1 | *Prunus avium* | Roberts, 2012 | e |
| R2-leaf/MH001 | *P. syringae* pv *morsprunorum* R2 | *Prunus avium* | Hulin, 2014 | abcdefgi |
| R2-5255 | *P. syringae* pv *morsprunorum* R2 | *Prunus avium* | Prunier, n.d. | e |
| R2-5260 | *P. syringae* pv *morsprunorum* R2 | *Prunus avium* | Garrett, n.d. | e |
| R2-7968A | *P. syringae* pv *morsprunorum* R2 | *Prunus avium* (wild) | Vicente, 2000 | e |
| R2-9095 | *P. syringae* pv *morsprunorum* R2 | *Prunus avium* (wild) | Roberts, 2010 | e |
| R2-SC214 | *P. syringae* pv *morsprunorum* R2 | *Prunus avium* | Roberts, 1983 | e |
| avii5271 | *P. syringae* pv *avii* | *Prunus avium* (wild) | Garrett, 1990 | ce |
| Pss-5275 | *P. syringae* pv *syringae* PG:2d | *Prunus avium* (wild) | Garrett, 1990 | e |
| Pss-9097 | *P. syringae* pv *syringae* PG:2d | *Prunus avium* | Roberts, 2010 | e |
| Pss-9293 | *P. syringae* pv *syringae* PG:2b | *Prunus domestica* | Roberts, 2011 | e |
| Pss-9644 | *P. syringae* pv *syringae* PG:2d | *Prunus avium* | Roberts, 2012 | abcdefgi |
| Pss 9656 | *P. syringae* pv *syringae* PG:2b | *Prunus avium* | Roberts, 2012 | e |
| Pss 9659 | *P. syringae* pv *syringae* PG:2d | *Prunus avium* | Roberts, 2012 | e |
| RMA1* | *P. syringae* sp. | *Aquilegia vulgaris* | Jackson, 2012 | i |
