# Supplementary material for: Identifying resistance in wild and ornamental cherry towards bacterial canker caused by Pseudomonas syringae
Source: Plant Pathol. 2021 Dec 21;71(4):949–65. doi: 10.1111/ppa.13513 (PMC9305585; doi:10.1111/ppa.13513)
Supplement: Supplementary file 4 — Table S3 [file PPA-71-949-s001.docx]

**Table S3**. Differential reactions recorded in leaves, grouped on resistance or susceptibility to strains based on the upper box plot line being greater than 1.0. The tabulated scores are for 0, box plot quartile less than 1; 1, 1-2; 2, 2-3 and 3 more than 3. Examples of clear differentials are highlighted in red. Note accession 21 which is resistant to all strains except *Psm*R1-P from plum.

| **Accession** | ***Psa*** | ***Psm* R1 -C** | ***Psm* R1-P** | ***Psm* R2** | ***Pss*** |
| --- | --- | --- | --- | --- | --- |
| **Resistant to *Psm R1*-C** |  |  |  |  |  |
| 9 | 0 | 0 | 1 | 2 | 1 |
| 21 | 0 | 0 | 3 | 0 | 0 |
| 20 | 0 | 0 | 0 | 0 | 2 |
| 42 | 0 | 0 | 0 | 1 | 3 |
|  |  |  |  |  |  |
| **Resistant to *Pss*** |  |  |  |  |  |
| 50 | 1 | 1 | 0 | 0 | 0 |
| 43 | 0 | 1 | 0 | 0 | 0 |
| 47 | 0 | 1 | 0 | 0 | 0 |
| 7 | 1 | 1 | 0 | 0 | 0 |
| 21 | 0 | 0 | 3 | 0 | 0 |
| 27 | 3 | 1 | 0 | 0 | 0 |
| 1 | 1 | 2 | 0 | 0 | 0 |
| 48 | 1 | 1 | 0 | 0 | 0 |
| 31 | 0 | 3 | 3 | 3 | 0 |
|  |  |  |  |  |  |
| **Resistant to *Psm* R2** |  |  |  |  |  |
| 50 | 1 | 1 | 0 | 0 | 0 |
| 43 | 0 | 1 | 0 | 0 | 0 |
| 47 | 0 | 1 | 0 | 0 | 0 |
| 7 | 1 | 1 | 0 | 0 | 0 |
| 21 | 0 | 0 | 3 | 0 | 0 |
| 27 | 3 | 1 | 0 | 0 | 0 |
| 1 | 1 | 2 | 0 | 0 | 0 |
| 20 | 0 | 0 | 0 | 0 | 2 |
| 13 | 0 | 1 | 2 | 0 | 3 |
| 48 | 1 | 1 | 0 | 0 | 0 |
|  |  |  |  |  |  |
| **Resistant to *Psa*** |  |  |  |  |  |
| 43 | 0 | 1 | 0 | 0 | 0 |
| 47 | 0 | 1 | 0 | 0 | 0 |
| 9 | 0 | 0 | 1 | 2 | 1 |
| 21 | 0 | 0 | 3 | 0 | 0 |
| 20 | 0 | 0 | 0 | 0 | 2 |
| 12 | 0 | 1 | 1 | 1 | 2 |
| 13 | 0 | 1 | 2 | 0 | 3 |
| 51 | 0 | 2 | 1 | 1 | 1 |
| 42 | 0 | 0 | 0 | 1 | 3 |
| 31 | 0 | 3 | 3 | 3 | 0 |
| 24 | 0 | 3 | 0 | 2 | 3 |
| 49 | 0 | 2 | 3 | 1 | 2 |
| 15 | 0 | 2 | 2 | 2 | 2 |
| 29 | 0 | 2 | 0 | 2 | 3 |
| 2 | 0 | 3 | 3 | 3 | 3 |
| 30 | 0 | 3 | 3 | 2 | 3 |
|  |  |  |  |  |  |
| **Resistant to *Psm* R1-plum** |  |  |  |  |  |
| 50 | 1 | 1 | 0 | 0 | 0 |
| 43 | 0 | 1 | 0 | 0 | 0 |
| 47 | 0 | 1 | 0 | 0 | 0 |
| 7 | 1 | 1 | 0 | 0 | 0 |
| 27 | 3 | 1 | 0 | 0 | 0 |
| 1 | 1 | 2 | 0 | 0 | 0 |
| 20 | 0 | 0 | 0 | 0 | 2 |
| 42 | 0 | 0 | 0 | 1 | 3 |
| 48 | 1 | 1 | 0 | 0 | 0 |
| 24 | 0 | 3 | 0 | 2 | 3 |
| 29 | 0 | 2 | 0 | 2 | 3 |
| 28 | 2 | 2 | 0 | 1 | 1 |
| 18 | 1 | 1 | 0 | 1 | 3 |
| 17 | 2 | 2 | 0 | 2 | 3 |
| 37 | 2 | 2 | 0 | 2 | 3 |
| 38 | 3 | 2 | 0 | 1 | 1 |
| 11 | 3 | 2 | 0 | 3 | 3 |
| 25 | 3 | 3 | 0 | 2 | 3 |
| 44 | 2 | 1 | 0 | 2 | 2 |
| 40 | 1 | 3 | 0 | 1 | 2 |
| 39 | 2 | 2 | 0 | 2 | 1 |
| 22 | 1 | 3 | 0 | 3 | 3 |
|  |  |  |  |  |  |
| **Susceptible to *Psm* R1-plum** |  |  |  |  |  |
| 21 | 0 | 0 | 3 | 0 | 0 |
| 12 | 0 | 1 | 1 | 1 | 2 |
| 13 | 0 | 1 | 2 | 0 | 3 |
| 51 | 0 | 2 | 1 | 1 | 1 |
| 31 | 0 | 3 | 3 | 3 | 0 |
| 49 | 0 | 2 | 3 | 1 | 2 |
| 15 | 0 | 2 | 2 | 2 | 2 |
| 2 | 0 | 3 | 3 | 3 | 3 |
| 4 | 1 | 2 | 3 | 2 | 3 |
| 30 | 0 | 3 | 3 | 2 | 3 |
| 8 | 1 | 3 | 3 | 3 | 3 |
|  |  |  |  |  |  |
